# Description of call handling in emergency medical dispatch centres in Scandinavia: recognition of out-of-hospital cardiac arrests and dispatcher-assisted CPR

**DOI:** 10.1186/s13049-021-00903-4

**Published:** 2021-06-30

**Authors:** Camilla Hardeland, Andreas Claesson, Marieke T. Blom, Stig Nikolaj Fasmer Blomberg, Fredrik Folke, Jacob Hollenberg, Jo Kramer-Johansen, Freddy Lippert, Anette Nord, Anne Mette Nygaard, Theresa Mariero Olasveengen, Mattias Ringh, Leif Svensson, Thea Palsgaard Møller

**Affiliations:** 1 Department of Health and Welfare, Østfold University College, P.O. box 700, NO-1757 Halden, Norway; 2 Norwegian National Advisory Unit on Prehospital Emergency Medicine (NAKOS), Division of Prehospital Services, Oslo University Hospital and University of Oslo, Oslo, Norway; 3 Department of Clinical Science and Education, Södersjukhuset, Centre for Resuscitation Science, Karolinska Institutet, Stockholm, Sweden; 4 Department of Cardiology, Heart Centre, Amsterdam UMC, Academic Medical Centre, University of Amsterdam, Amsterdam, The Netherlands; 5 Copenhagen Emergency Medical Services, University of Copenhagen, Copenhagen, Denmark; 6 Department of Anesthesiology, Oslo University Hospital, Oslo, Norway; 7 Department of Medicine, Centre for Resuscitation Science, Karolinska Institutet, Stockholm, Sweden

**Keywords:** Emergency medical dispatch, Cardiac arrest, Cardiopulmonary resuscitation, Cpr, Emergency medical dispatch Centre, Dispatcher, Out-of-hospital cardiac arrest

## Abstract

**Background:**

The European resuscitation council have highlighted emergency medical dispatch centres as an important key player for early recognition of Out-of-Hospital Cardiac Arrest (OHCA) and in providing dispatcher assisted cardiopulmonary resuscitation (CPR) before arrival of emergency medical services. Early recognition is associated with increased bystander CPR and improved survival rates. The aim of this study is to describe OHCA call handling in emergency medical dispatch centres in Copenhagen (Denmark), Stockholm (Sweden) and Oslo (Norway) with focus on sensitivity of recognition of OHCA, provision of dispatcher-assisted CPR and time intervals when CPR is initiated during the emergency call (NO-CPR_prior_), and to describe OHCA call handling when CPR is initiated prior to the emergency call (CPR_prior_).

**Methods:**

Baseline data of consecutive OHCA eligible for inclusion starting January 1st 2016 were collected from respective cardiac arrest registries. A template based on the Cardiac Arrest Registry to Enhance Survival definition catalogue was used to extract data from respective cardiac arrest registries and from corresponding audio files from emergency medical dispatch centres. Cases were divided in two groups: NO-CPR_prior_ and CPR_prior_ and data collection continued until 200 cases were collected in the NO-CPR_prior_-group.

**Results:**

NO-CPR_prior_ OHCA was recognised in 71% of the calls in Copenhagen, 83% in Stockholm, and 96% in Oslo. Abnormal breathing was addressed in 34, 7 and 98% of cases and CPR instructions were started in 50, 60, and 80%, respectively. Median time (mm:ss) to first chest compression was 02:35 (Copenhagen), 03:50 (Stockholm) and 02:58 (Oslo). Assessment of CPR quality was performed in 80, 74, and 74% of the cases. CPR_prior_ comprised 71 cases in Copenhagen, 9 in Stockholm, and 38 in Oslo. Dispatchers still started CPR instructions in 41, 22, and 40% of the calls, respectively and provided quality assessment in 71, 100, and 80% in these respective instances.

**Conclusions:**

We observed variations in OHCA recognition in 71–96% and dispatcher assisted-CPR were provided in 50–80% in NO-CPR_prior_ calls. In cases where CPR was initiated prior to emergency calls, dispatchers were less likely to start CPR instructions but provided quality assessments during instructions.

**Supplementary Information:**

The online version contains supplementary material available at 10.1186/s13049-021-00903-4.

## Background

Out-of-Hospital Cardiac Arrest (OHCA) affects approximately 350,000 people in Europe and 700,000 in the United States each year [1, 2]. Incidence rates in Europe varies from 27 to 91 per 100,000 population per year [3]. The European resuscitation council (ERC) have highlighted emergency medical dispatch centres (EMDCs) as an important key player for early recognition of OHCA and in providing dispatcher assisted cardiopulmonary resuscitation (DA-CPR) before emergency medical services (EMS) arrival [1]. Early recognition of OHCA is associated with early and increased rates of bystander cardiopulmonary resuscitation (CPR) and improved survival rates [4–8]. Implementation of scripted protocols are associated with improved recognition of OHCA by emergency medical dispatchers (EMDs) [1, 5, 9] who handle and prioritize the emergency calls. Performance goals for recognition of OHCA in EMDCs are not well established, but it is suggested that EMDCs should aim to recognise 95% of all OHCA cases in calls where the dispatcher is able to assess consciousness and breathing. Further, recognition should be established within 1 min from the start of a call, and DA-CPR initiated within 2 min [10–12].

Recognition sensitivity of OHCA differ between countries and dispatch centres, with reported numbers ranging from 14 to 97%, median 74% [13]. This variation may be due to the difference in EMS organisations or the definition of recognition of OHCA in the reported data. Another limitation in the comparison between existing studies may be the inclusion criteria for the OHCAs. A difference in the context of the OHCA exists and thus the premise for recognition. For example, cardiac arrests in wich bystander are already undergoing CPR at the time of the emergency call differs from OHCA where CPR is not initiated prior to the call in terms of recognition, given the bystanders awareness of the OHCA. A study has disproved the association between bystander CPR initiation before the emergency call and survival, despite the fact that the OHCA is already recognized by bystander prior to the emergency call [14]. We speculate that this lack of association illustrates a more efficient resuscitation attempt if the medical diaspatchers guide bystanders throughout the process, from recognition of the OHCA to ambulance arrival. In a study combining closed-circuit television and medical emergency calls, Linderoth et al. discovered poor quality CPR in some cases where bystanders started CPR on their own initiative [15].

Despite the high proportion of emergency calls in which bystander CPR has begun prior to the emergency call (reported as high as 35%) [14], and despite the potential for improvement of CPR quality, little is known about callhandling in these specific emergency calls. In studies focusing on performance of DA-CPR, cases in which bystander CPR is initiated prior to the call is often excluded [5, 16, 17] and current ERC guidelines [1] lack guidance about DA-CPR in this context.

The aim of this study is to: (1) describe OHCA call handling in EMDCs in three Scandinavian capitals with special focus on sensitivity of recognition of OHCA, provision of dispatcher-assisted CPR and time intervals and (2) to describe OHCA call handling in cases where CPR is initiated prior to the emergency call.

## Methods

### Study design and setting

This is an observational, multicentre study including dispatch centres in capital regions of the Scandinavian countries: Copenhagen (Denmark), Stockholm (Sweden) and Oslo (Norway). All three study sites used criteria based dispatch (CBD). In a CBD system, dispatchers rely on a decision support tool in addition to their own knowledge and experience. All sites used local adjusted versions of the third Norwegian index for emergency care [18] which instruct dispatchers to verify unconsciousness before proceeding to the protocol for “unconscious patient, not breathing normally”. How to establish abnormal breathing is an individual assessment, consequently some dispatchers ask if the patient is breathing (breathing addressed), while others ask if the patient is breathing normally (abnormal breathing also addressed). The protocol further provides a structured dialogue describing CPR instructions. Repeated assessments of quality of bystander-CPR or techniques to motivate and encourage the bystander while performing CPR are not described as part of the protocol, and dispatchers make individual decisions on measures to ensure ongoing CPR of high quality. The only difference in protocol between sites, was that in Copenhagen, they ask whether or not the caller is skilled in CPR, but in Stockholm and Oslo this question was not part of the protocol. Coordinated public access Automated External Defibrillation (AED) programs were implemented in and linked electronically to the dispatch prioritization tool in Copenhagen and Stockholm, but not in Oslo. Study site characteristics are shown in Table 1.

**Table 1:** Study site characteristics (2016)

	Greater Copenhagen Denmark	Stockholm region Sweden	Oslo region Norway
Population	1.8 million	2.3 million	1.6 million
Area covered by EMDC	2568 km^2^	6519 km^2^	9551 km^2^
Incidence of OHCA	82/100,000	45/100,000	61/100,000
Incoming emergency calls 2016	133,772	206,729	172,934
Proportion of calls resulting in an ambulance response	Priority 1: 42,645 (32%) Priority 2: 41,528 (31%) Priority 3 (non-emergency): 28,281 (21%)	Priority 1: 115,453 (56%) Priority 2: 80,716 (39%) Priority 3 (non-emergency): 10,567 (5%)	Priority 1: 77,460 (45%) Priority 2: 70,269 (41%) Priority 3 (non-emergency): 45,724 (26%)
Number for emergency	112 for medical, police, and fire/rescue	112 for medical, police, and fire/rescue	113 for medical, 112 for police, and 110 for fire/rescue
Medical dispatcher background	Nurse / paramedic	Nurse/Other	Nurse/paramedic
Specific training in handling cardiac arrest calls in the dispatch centre.	Yes	Yes	Yes
Manual/electronic use of Index	Electronic	Electronic	Manual

#### Copenhagen

Copenhagen EMS has one EMDC covering the largest of five regions in Denmark. The emergency phone number 1–1-2 connects to a primary call centre that locates the address and categorises the need for police, fire department or medical assistance. In case of a medical emergency, the call is forwarded to an EMDC that answers the call, reconfirms the address and responds by activating the appropriate EMS response. The medical dispatchers are registered nurses (RN) (70%) or paramedics (30%) with 6 weeks of additional training in communication and the prioritization of emergency calls.

#### Stockholm

The EMDC in Stockholm is one of 15 nationally linked EMDCs. Dispatchers are obliged to answer 112-calls within 15 s in 92% of all cases. In In high call volume periods, 112-calls are automatically transferred to free dispatchers in other EMDCs throughout Sweden in order to reduce time delay in answering calls. In 2016, 20% of the staff at the EMDC in Stockholm were RN’s whilst 80% were nursing assistants or non health care providers. The training consisted of two blocks, a) 13 weeks of theory and practice followed by b) 8 weeks of supervised work. Annual re-certification is generally required for all EMDs.

#### Oslo

Oslo EMDC is the largest in Norway and covers both urban and rural areas. The EMDC is staffed with 46% emergency medical technicians (EMTs)/paramedics (EMTs with 6 months further education) coordinating ambulance responses and 54% RNs (EMDs) answering emergency calls. Training consist of 4 weeks of lectures/theory, then approximately 2 months working under supervision of another EMD. Time spent under supervision depends on the individuals progress and prior experience.

### Data collection

**Fig. 1:**
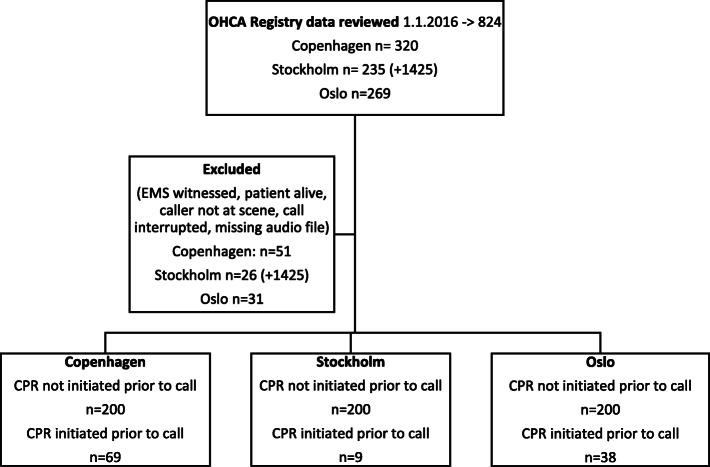
Inclusion strategy

We identified OHCAs from the respective national cardiac arrest registries and linked the cases with data from the EMDC to obtain the audio files of the emergency calls. Inclusion criteria in the cardiac arrest registries were the same in each country; CPR initiated by either bystander or EMS. The data collection period was from January 1st, 2016 to March 17th, 2016 (Copenhagen), March 24th 2017 (Stockholm) and May 12th, 2016 (Oslo). We excluded OHCA characterized as: (1) cardiac arrests witnessed by EMS personnel (“EMS witnessed”), (2) patient alive at time of call, (3) caller cannot access patient, (4) call interrupted, and (5) audio file not available. Cases were divided in two groups: In group 1 (referred to as NO-CPR_prior_) CPR was NOT initiated prior to the emergency call, in group 2 (referred to as CPR_prior_), CPR was initiated by the bystander prior to the emergency call. Consecutive OHCAs were collected until 200 OHCA cases in the NO-CPR_prior_ group was included from each study site. Totally 200 cases from each site was deemed sufficient to provide base line characteristics in accordance with our aim. Both data collection period and number of included cases in CPR_prior_-group varied in the three study sites (Fig. 1).

In Stockholm, a large number of calls are handled by other EMDCs and were excluded before review (*n* = 1425). There is a national dispatch organisation in Sweden where all calls can be handled in either of 15 different dispatch centres. This has no effect on delays or quality of DA-CPR instructions, but collection of calls from Stockholm and handled by the Stockholm dispatch centres was therefore extended in time.

A common template in combination with a detailed data dictionary was used when reviewing OHCA calls. The template and data dictionary were based on the “Cardiac Arrest Registry to Enhance Survival” (CARES) data dictionary [19], with some adjustments, see (Additional file 1) for the complete data dictionary. A total of 34 data points were collected from the audio files, focusing on recognition of cardiac arrest, time intervals, and provision of DA-CPR. Several meetings with the reviewers were held to discuss data points, inclusion criteria, terminology/definitions and difficult cases. Data collection was performed by three researchers in Copenhagen, three in Stockholm and two in Oslo. Cardiac arrest was defined as recognised if the dispatcher indicated that CPR should be performed during the call. We also registered whether the dispatcher made assessment of quality of performance of bystander-CPR during the calls, such as «please count out loud with me», «are you pushing deep enough?», «push a bit faster/slower» and if dispatchers used encouraging and motivational techniques, e.g. «keep on going», «you’re doing a great job», «the ambulance is on its way».

Data points from cardiac arrest registries which were similar across all three sites were collected, such as patient characteristics, witness status, initial rhythm and ambulance treatment.

### Statistical analysis

Descriptive analysis were performed using a spreadsheet program (Microsoft Excel 2019, Microsoft Corp, Redmond, WA, USA) or a statistical software package (SPSS 26.0, SPSS Inc., Chicago, IL, USA). Values are provided as numbers with percentages or medians with interquartile range. Categorical data were analysed using Pearson chi-squared test. Comparisons of continuous data were done with non-parametric Independent-Samples Kruskal-Wallis Test. *P*-Values ≤0.05 were considered significant.

## Results

In order to include 200 OHCA cases from each country (NO-CPR_prior_), 320 calls were reviewed in Copenhagen, 235 in Stockholm, and 269 in Oslo. Cases in which CPR was initiated prior to call (CPR_prior_) comprised 69 cases in Copenhagen, 9 in Stockholm, and 38 in Oslo (Fig. 1). An overview of patient and resuscitation characteristics are shown in Table 2, and dispatcher performance is shown in Table 3.

**Table 2:** Patient and resuscitation characteristics of OHCA call handling

**Group 1: No CPR prior to call**	**Copenhagen (*N*** **= 200)**	**Stockholm (*N*** **= 200)**	**Oslo (*N*** **= 200)**
Age (years) median [IQR]	72 [62;82]	72 [59; 82]	65 [47; 77]
Unknown	–	4 (2)	–
Male gender	121 (61)	122 (61)	129 (65)
Unknown	9 (5)	1 (1)	–
Location			
Home	157 (79)	152 (76)	142 (71)
Public	19 (10)	32 (16)	42 (21)
Other	24 (12)	16 (8)	16 (8)
Caller is health care personnel	45 (23)	30 (15)	39 (20)
Unknown	–	47 (24)	6 (3)
Bystander witnessed	106 (53)	105 (53)	103 (52)
	–	5 (3)	–
Bystander CPR	130 (65)	109 (55)	183 (92)
	–	–	4 (2)
Bystander defibrillation (AED)	14 (7)	5 (3)	8 (4)
Initial shockable rhythm	33 (17)	32 (16)	33 (17)
CPR by EMT	179 (90)	189 (95)	138 (69)
Defibrillation by EMT	47 (24)	64 (33)	45 (23)
**Group 2: CPR initiated prior to call**	**Copenhagen (*N*** **= 69)**	**Stockholm (*N*** **= 9)**	**Oslo (*N*** **= 38)**
Age (years) median [IQ1;IQ3]	75 [60; 86]	66 [62; 78]	66 [44; 79]
Male gender	31 (45)	7 (78)	26 (68)
Unknown	3 (4)	–	–
Location			
Home	41 (59)	2 (22)	13 (34)
Public	7 (10)	4 (44	13 (34)
Other	21 (30)	3 (33)	12 (32)
Caller is health care personnel	39 (57)	4 (44)	17 (45)
Unknown	–	4 (44)	–
Bystander witnessed	30 (43)	4 (44)	24 (63)
Bystander defibrillation (AED)	16 (23)	1 (11)	4 (11)
Initial shockable rhythm	11 (16)	2 (22)	10 (26)
CPR by EMT	56 (81)	8 (89)	26 (68)
Defibrillation by EMT	14 (20)	5 (63)	11 (29)
Unknown	–	1 (11)	–

Values given as numbers (percentages) *Abbreviations*: *CPR* cardiopulmonary resuscitation, *IQR* Inter quartile range, *AED* automated external defibrillator, *EMT* Emergency medical technician

**Table 3:** Dispatcher performance

**Group 1: No CPR prior to call (CPR**_**during**_**)**	**Copenhagen (*N*** **= 200)**	**Stockholm (*N*** **= 200)**	**Oslo (*N*** **= 200)**	***p*-value**
Consciousness addressed	168/200 (84)	189 /200 (95)	199/200 (100)	*p* = 0.001
Unknown	17/200 (9)	–	–	
Breathing addressed	169/200 (85)	196 /200 (98)	199/200 (100)	*p* < 0.001
Unknown	19/200 (10)	–	–	
Abnormal breathing addressed	68/200 (34)	13/200 (7)	195/200 (98)	*p* < 0.001
Unknown	15/200 (8)	1/200 (1)	–	
OHCA recognition	142/200 (71)	165/200 (83)	192/200 (96)	*p* < 0.001
Unknown	13/200 (7)	–	1/200 (1)	
CPR instructions started	99/200 (50)	120/200 (60)	160/200 (80)	*p* < 0.001
Unknown	24/200 (12)	–	–	
Dispatcher is assertive when providing CPR instructions	84/99 (85)	101/120 (84)	145/160 (91)	*p* = 0.2
Unknown	–	3/120 (3)	–	
Quality assessment	79/99 (80)	89/120 (74)	119/160 (74)	*p* = 0.2
Unknown	–	7/120 (6)	–	
Encouraging/motivating techniques in use	69/99 (70)	99/120 (83)	132/160 (83)	*p* < 0.001
Chest compressions performed	109/200 (55)	119/200 (60)	157/200 (79)	*p* < 0.001
Unknown	–	5/200 (3)	4/200 (2)	
Type of CPR				*p* = 0.3
30:2	24/109 (22)	30/119 (25)	46/157 (29)	
Compressions only	85/109 (78)	77/119 (65)	102/157 (65)	
Unknown	–	20/119 (17)	9/157 (6)	
BLS competence addressed	88/200 (44)	101/200 (51)	63/200 (32)	*p* < 0.001
Unknown	30/200 (15)	1/200 (1)	–	
AED addressed	22/200 (11)	8/200 (4)	6/200 (3)	*p* < 0.001
Unknown	51/200 (26)	–	–	
Call continued until EMS arrival	85/200 (43)	128/200 (64)	163/200 (82)	*p* < 0.001
Unknown	14/200 (7)	–	3/200 (2)	
Time intervals for recognition and CPR instructions				
Time to OHCA recognition (min:sec)	01:16 [IQR 00:50–02:11]	01:53 [IQR 1:01–3:13]	01:19 [IQR 0:50–2:09]	*p* < 0.001
Time to chest compression instructions (min)	02:10 [IQR 01:27–03:25]	03:20 [IQR 02:03–04:56]	02:24 [IQR 01:37–04:00]	*p* < 0.001
Time to chest compressions performed (min)	02:35 [IQR 01:45–03:05]	03:50 [IQR 02:30–05:27]	02:58 [IQR 02:09–04:36]	*p* < 0.001
**Group 2: CPR initiated prior to call (CPR**_**prior**_**)**	**Copenhagen (*N*** **= 69)**	**Stockholm (*N*** **= 9)**	**Oslo (*N*** **= 38)**	
CPR instructions started	28/69 (41)	2/9 (22)	15/38 (40)	
Unknown	8/69 (12)	–	–	
Dispatcher is assertive when providing CPR instructions	24/28 (86)	2/2 (100)	15/15 (100)	
Quality assessment	20/28 (71)	2/2 (100)	12/15 (80)	
Encouraging/motivating techniques in use	20/28 (71)	2/2 (100)	14/15 (93)	
Type of CPR				
30:2	17/69 (25)	1/9 (11)	7/38 (18)	
Compressions only	30/69 (43)	1/9 (11)	12/38 (32)	
Unknown	22/69 (32)	7/9 (78)	19/38 (50)	
AED addressed	27/69 (39)	3/9 (33)	4/38 (11)	
Call continued until EMS arrival	25/69 (36)	4/9 (44)	19/38 (50)	

Categorical data are presented as numbers (percentages) and were analysed using Pearson chi-squared test. Continuous data are presented as medians (interquartile range) and compared with non-parametric Independent-Samples Kruskal-Wallis test

In the NO-CPR_prior_ group, OHCA was recognised during the call in 71% of the cases in Copenhagen, 83% in Stockholm and 96% in Oslo. CPR instructions were started in 50, 60, and 80%, respectively (Table 3). CPR instructions were not started in cases were cardiac arrest was not recognised, caller was unable or unwilling to perform CPR, or caller was not at scene. ‘In cases where CPR instructions were given, Quality assessment was used in 80, 74, and 74% of the cases. Encouraging and/or motivating techniques were in use in 70, 83 and 83% respectively. Median time (mm:ss) to recognition of OHCA was 01:16 (Copenhagen), 01:53 (Stockholm) and 01:19 (Oslo) and median time to first chest compression was 02:35 (Copenhagen), 03:50 (Stockholm) and 02:58 (Oslo).

In the CPR_prior_ group, dispatchers started CPR instructions in 41% of the cases in Copenhagen, 22% in Stockholm, and 40% in Oslo. In cases where instructions were started, quality assessment was used in 71, 100, and 80% of the cases. Encouraging and/or motivating techniques were in use in 71, 100 and 93% respectively.

Across all three sites, the NO-CPR_prior_ and CPR_prior_ group comprised 600 versus 116 cases, respectively. CPR instructions were started in 63% in NO-CPR_prior_ and 39% in CPR_prior_. In cases were CPR instructions were started, quality assessment and encouraging and/or motivational techniques were similarly used in the two groups. Quality assessment was found in 76% of cases in both groups, and encouraging and/or motivational techniques were found in 79% of the cases in NO-CPRprior and 80% in the CPRprior group. The caller was a health care personnel in 19% in NO-CPR_prior_ and 52% in CPR_prior_ group.

## Discussion

The main results of this study show that OHCA recognition rates in cases were no CPR was initiated prior to call varied from 71 to 96% between three capital city EMDCs in Scandinavia. Time to recognition varied by over half a minute, and time to first chest compression varied by over a minute. We question whether this is due to differences in dispatcher performance or differences in systems as system variables were difficult to compare objectively.

### OHCA recognition

International literature shows extensive variations in recognition sensitivity of OHCA. 13]. Reasons for the significant difference in OHCA recognition in this study is multifaceted. Oslo had 1 ½ years prior to this study undergone a targeted intervention to improve performance in recognition rates and DA-CPR [20]. ‘Attention to appropriate handling of cardiac arrest calls was an ongoing priority in the EMDC in 2016, which may have affected the high level of recognition in Oslo. The ERC guidelines 2015 state that dispatchers can improve recognition by focusing on “unresponsiveness” and “not breathing normally” [1]. All three sites addressed consciousness and breathing in most cases, but Oslo was the only site also addressing abnormal breathing in most cases. This might be the main reason for differences in recognition rates between the three countries.

Differences in educational levels or professional background of the dispatchers might also be factors impacting on recognition rates. In Stockholm, EMDs were not necessarily health care personnel, and only 20% were nurses. In Oslo and Copenhagen, EMDs were nurses or paramedics.

Dispatcher performance can directly affect OHCA recognition rates by including false OHCA cases in the cardiac arrest registry. Inclusion criteria in the cardiac arrest registries in the Scandinavian countries are the same; all cases where anyone at scene (bystander or EMTs) have started CPR, is included in the registry. But if callers are instructed to start CPR in unclear cases where the patient is actually not in cardiac arrest, they are still included in the registry. Hence, pro-active dispatchers can increase the incidence numbers in the cardiac arrest registries by providing unnecessary CPR instructions. Although not reported in this study, these cases might also affect outcome data, possibly including both cases were the patient was never in cardiac arrest, as well as cases where no treatment was started by EMT due to futility.

Despite a high proportion of recognised cases in all three sites, and AEDs mentioned in the protocol, AEDs were rarely addressed in the NO-CPR_prior_ group. Reasons for this is unknown, but a study from Sweden exploring this specifically found that AEDs were not nearby in 93% of the cases. Other reasons might be inaccessible AEDs and caller being alone [21]. There were significant differences between sites on AED addressed in this study (11% in Copenhagen, 4% in Stockholm and 3% in Oslo). Longstanding efforts from the Danish AED registry might have affected this. Oslo did not have a functional AED registry at the time of data collection.

There was seemingly a relatively long time interval from OHCA recognition to start of first chest compression instruction in all sites (00,54 in Copenhagen, 01:27 in Stockholm, 01:05 in Oslo). This was due to the Cares definition of time interval for “chest compression instruction”, which states that “Instructions to get a patient to a hard, flat surface should not be considered the start of CPR instructions. Instructions begin when a call-taker or dispatcher tells the rescuer to “kneel by the patient’s side.”” [19].

### CPR initiated prior to the emergency call

There were great variations in proportions of cases were CPR was initiated prior to call in the three study sites (69 (Copenhagen) versus 9 (Stockholm) versus 38 (Oslo)). Reasons for this might be differences in basic life support (BLS) programs or organisational differences, in Copenhagen all calls are handled by the police before transferred to a medical dispatcher, providing more time for qualified bystanders to initiate CPR. Results from this study indicate that dispatchers are less likely to provide CPR instructions to callers when CPR is initiated prior to the emergency call. Early CPR is associated with increased survival [22–24] but favourable outcome depends on high quality CPR performance [25]. Studies comparing DA-assisted CPR and bystander initiated CPR prior to the call have found no significant difference in survival between the two groups [13, 26]. However, Takei et al. showed significantly more good quality CPR compared to low quality CPR (OR 2.67) in bystander-initiated CPR prior to the call to the EMDC [27]. It is fair to assume that bystanders who start CPR without instructions are at least willing to perform CPR. Their skills and abilities are uncertain at the time of the emergency call and might be clarified by the medical dispatchers who in addition can support the bystander in performing high quality CPR. Lack of CPR instructions to bystanders performing CPR prior to call indicates a knowledge gap. There is a need for further exploration of the consequences when dispatchers do not provide CPR instructions to a large group of bystanders in a group mostly excluded from studies on DA-CPR.

A high proportion of health care personnel as callers might explain why dispatchers to a lesser extent provide CPR instructions in cases where CPR is initiated prior to the call. Health care professional bystanders are more likely to initiate CPR prior to the call [13]. CPR performed by health care professionals has been shown to have increased patient survival compared to bystander initiated CPR performed by laypeople [28, 29], but a recent study showed no such difference [30]. It can be difficult for dispatchers to assess callers’ competence in CPR based on the fact that they are health care providers. For example, a common situation in OHCA is that the patient is discovered by representatives from the home care services, and the caller is perceived by the dispatcher to be a health care provider. The home care services can be staffed by people with no or limited medical training [31] and potentially no experience in handling a cardiac arrest patient, hence in as much need of CPR instructions as lay people.

When CPR is initiated prior to the call to the EMDC, cases are recommended to be excluded from review in studies reporting OHCA recognition rates, [13, 17]. These cases are rarely described, even though this seems necessary in order to give proper recommendations for dispatchers on handling these cases. In the future it is reasonable to include more information on the interaction in the first resuscitation team in BLS-programs. Likewise, terminology should be standardised between national CPR councils providing course curriculums and dispatch organisations decision and prioritisation tools, also in cases where CPR is initiated prior to emergency calls. To avoid unnecessary complications and ensure optimal treatment of all OHCA patients, we suggest that dispatchers always provide CPR instructions (also to health care personnel and when CPR is initiated prior to call), make quality assessments during the call and stay on the line until EMS arrival.

### Limitations

This study has several limitations. There are few cases where CPR is initiated prior to the call, and the numbers vary between the three study sites. More data would have made comparisons possible. These data should therefore be regarded as explorative findings providing hypothesis generating knowledge on a group rarely described in other studies. A low number of cases from each site and potential differences in BLS-programs may interfere with the analysis. Inclusion and outcome data is affected by dispatcher performance between sites, and differences in system organisations make comparisons between sites difficult. For example OHCA incidence (per 100,000 inhabitants) in the registries differ from 45 (Stockholm), 61 (Oslo) and 85 (Copenhagen), and CPR by EMT is lower in Oslo than Stockholm and Copenhagen. There is no reason to believe that there are great variations in morbidity between countries, and explanations to these variations might be found in inclusion criteria and reporting rate to the cardiac arrest registries. Time differences between sites are also likely to differ due to differences in call handling. In Denmark the emergency call is initially answered by the police who then refers the call to the EMDC, time variable is measured from EMDC taking the call. In Stockholm the EMDC answer all emergency calls, and 70% of calls are not medical emergencies. In Oslo there is a specific telephone number for medical emergencies only.

High recognition rate may be associated with a higher false positive rate. This is important because of scarce resources which influences management decisions. We have not been able to establish a false positive rate in this study. When reviewing audio files there will always be some interpretation by the reviewer. We tried to minimize this issue by a detailed data extraction protocol. Despite meticulous preparations, there are cases not possible to determine accurately from audio files, resulting in more unknown cases than expected (Table 3). Reasons for this were most often if callers were excessively distraught, (in quite a few cases the caller left the phone) or there were language barriers. Reviewers from respective countries did not have access to other countries’ audio files, and no interrater agreement across countries could be performed.

## Conclusions

In this study, we used a common template to collect data from EMDCs with seemingly similar health systems in the three Scandinavian capital regions. However, the main findings show variations in OHCA recognition and provision of DA-CPR, and we question whether this is due to differences in dispatcher performance or differences in systems. Further exploration of reasons for these variations are necessary. Descriptions of calls where CPR was initiated prior to contacting the EMDCs indicate that callers are more likely to be health care personnel, and dispatchers are less likely to provide CPR instructions to such callers. Further studies to explore the consequences of not providing CPR instructions to bystanders are needed.

## Supplementary Information

**Additional file 1.**

## Data Availability

Not applicable.

## Abbreviations

AED: Automated External Defibrillator
CARES: Cardiac Arrest Registry to Enhance Survival
OHCA: Out-of-hospital cardiac arrest
ERC: The European resuscitation council
EMDC: Emergency medical dispatch centre
DA-CPR: Dispatcher assisted cardiopulmonary resuscitation
EMS: Emergency medical services
EMD: Emergency medical dispatcher
RN: Registered nurse
