# Supplementary material for: Description of call handling in emergency medical dispatch centres in Scandinavia: recognition of out-of-hospital cardiac arrests and dispatcher-assisted CPR
Source: Scand J Trauma Resusc Emerg Med. 2021 Jun 30;29:88. doi: 10.1186/s13049-021-00903-4 (PMC8247132; doi:10.1186/s13049-021-00903-4)
Supplement: Supplementary file 1 — Additional file 1. [file 13049_2021_903_MOESM1_ESM.docx]

**Dispatcher-Assisted**

**CPR Data Dictionary–**

**COSTA dispatch project**

Version 2.3

**02.05.18**

Data dictionary based on the “Cardiac Arrest Registry to Enhance Survival”

(CARES) Dispatcher-Assisted CPR Data Dictionary. 2015 <https://mycares.net/>)

Contents

[1. Call assessment necessary 4](#_Toc513216041)

[2. Audio file available 4](#_Toc513216042)

[3. Country 4](#_Toc513216043)

[4. Data collector initials 4](#_Toc513216044)

[5. Filename 4](#_Toc513216045)

[6. Time of dispatcher addressing caller (MM:SS) 4](#_Toc513216046)

[7. CPR already in progress? 5](#_Toc513216047)

[8. Consciousness addressed? 5](#_Toc513216048)

[9. Patient is conscious? 6](#_Toc513216049)

[10. Breathing addressed? 7](#_Toc513216050)

[11. Abnormal breathing adressed 7](#_Toc513216051)

[12. Patient is breathing normally? 8](#_Toc513216052)

[13. Did dispatcher recognise need for CPR? 9](#_Toc513216053)

[14. Delayed recognition due to deviation from cardiac arrest protocol 9](#_Toc513216054)

[15. Time of recognition (MM:SS) 10](#_Toc513216055)

[16. BLS competence addressed? 11](#_Toc513216056)

[17. CPR instructions started? 12](#_Toc513216057)

[18. Is the dispatcher assertive or passive when providing CPR instructions? 13](#_Toc513216058)

[19. Quality assessment 13](#_Toc513216059)

[20. Encouraging/motivating techniques in use 13](#_Toc513216060)

[21. Type of CPR 14](#_Toc513216061)

[22. Time of chest compression instructions started (MM:SS) 14](#_Toc513216062)

[23. Chest compressions started? 15](#_Toc513216063)

[24. Time of first chest compression (MM:SS) 16](#_Toc513216064)

[25. Was an AED addressed 17](#_Toc513216065)

[26. Was an AED connected to the patient? 17](#_Toc513216066)

[27. Did the AED deliver a shock to the patient? 17](#_Toc513216067)

[28. Barriers delaying or hindering CPR? 17](#_Toc513216068)

[29. Was CA witnessed by another person? 20](#_Toc513216069)

[30. Call continued until EMS arrival 20](#_Toc513216070)

[31. Caller gender 21](#_Toc513216071)

[32. Caller relation to patient 21](#_Toc513216072)

[33. Healthcare professional 21](#_Toc513216073)

[34. Was caller alone at time of call? 21](#_Toc513216074)

**Abbreviations**

AED - automated external defibrillator

BLS - Basic life support

CA - cardiac arrest

CC – Continous compressions

CPR - cardiopulmonary resuscitation

EMS - emergency medical services

N/A - not applicable

OHCA - out of hospital cardiac arrest

The following Data Dictionary defines the terms used on the Telephone CPR Data Form. Please review it carefully and refer to it as needed – it is essential to have a clear understanding of the elements the data form aims to collect. Coding these elements requires attention to detail and, in some cases, careful judgment, so evaluators should code a few sample cases before providing data to CDP.

**Exclusion criteria for assessment of calls**

- Patient alive at time of call
  - If the patient show undisputable signs of life, such as attempts to speak or move
  - If the OHCA is witnessed by EMS personell
- Caller access to assessment of patient
  - No close visual contact to the patient, can not see and/or touch the patient directly or by leaving the phone.
  - If access to the patient is impossible in the current situation.
  - When in doubt
- Call interrupted
  - If the call is interrupted before assesment of the patient is possible, and no recall can be identified
- Missing audio files

# Call assessment necessary

- Yes, If all inclusion criteria are met
- No, if one or more exclusion criteria are met

# Audio file available

- Yes
- No

# Country

- Norway
- Sweden
- Denmark

# Data collector initials

- Short answer text

# Filename

- Short answer text

**Definition:**

The individual file name assigned to each case from the cardiac arrest registry cases.

# Time of dispatcher addressing caller (MM:SS)

- Short answer text

# CPR already in progress?

- Yes - if CPR is known to have started by lay or trained bystanders on the scene before dispatcher starts instructions for CPR.
- No - If CPR is not started by lay or trained bystanders on the scene before dispatcher starts instructions for CPR.
- Unknown
- N/A

**Definition:**

 CPR is “already in progress” when callers indicate that they or other lay or trained rescuers on scene have started CPR before the dispatcher starts instructions for CPR.

**Description:**

 Calls where CPR is already in progress should be excluded when calculating the proportion of cases where dispatchers recognize the need for CPR, start CPR instructions, and achieve the first bystander compression. They should also be excluded when calculating the median or average time to these events from the start of the call.

**Instructions for Coding:**

 If CPR is known to start before a call-taker or dispatcher start instructions for CPR, mark the circle next to “Yes” under “CPR already in progress?”

 If CPR does not start before a call-taker or dispatcher starts instructions for CPR, mark the circle next to "No.”

 If it is not known whether CPR started before a call-taker or dispatcher started instructions for CPR, mark the circle next to “Unknown.”

 If “No” or “Unknown” is chosen, continue evaluating the call and completing the form.

 **If “Yes” is chosen, continue to code the following data variables: 17. CPR instructions started, 18. Is the dispatcher assertive or passive when providing CPR instructions? 19. Quality assessment, 20. Encouraging/motivating techniques in use and 21. Type of CPR.**

**25. Was an AED addressed? 26. Was an AED connected to the patient? 27. Did the AED deliver a shock to the patient? 28. Barriers delaying or hindering CPR? 29. Was CA witnessed by another person? 30. Call continued until EMS arrival 31. Caller gender 32. Caller relation to patient 33. Health care professional 34. Was caller alone at time of call?**

# Consciousness addressed?

- Yes
- No
- Unknown
- N/A
- Not relevant

1: If signs of consciousness is addressed by either dispatcher or caller (”awake”, ”conscious”, ”contact”, ”reactions” or similar terms). If the caller says that the patient is in cardiac arrest. Implications, such as “he is all gone” or “he collapsed” is not enough – it needs to be directly addressed. 2: if consciousness is not addressed. N/A: When in doubt. Not relevant: Dispatchers initiate CPR instructions without addressing consciousness. For example in cases where caller say that the patient is dead.

# Patient is conscious?

- Yes - If the caller reports that the patient is conscious, is responsive to the caller, or is making purposeful movement.
- No - If the patient can't speak, doesn't respond, is blurry, or won't wake up.
- Unknown
- N/A

**Definition:**

 A patient is considered conscious if the caller reports the patient is conscious and/or responsive to the caller. A patient is considered not conscious if the caller reports the patient is not conscious and/or is not responsive to the caller.

**Description:**

 A patient’s level of consciousness is a key indicator of whether he or she is in cardiac arrest. It can be difficult to get a clear answer on whether the patient is conscious. Callers often give contrary answers to this question at different times in the call. Type-appropriate CPR instructions should be given when a patient is deemed not conscious and not breathing normally.

**Instructions for Coding:**

 Mark the circle next to the appropriate answer (“Yes,” No,” or “Unknown”) under “Conscious?” on the “Dispatch: Patient” section of the QA form.

| **Examples:**  The caller says her husband is “passed out and not responding.” | Mark the “No” circle, coding the patient as not conscious. |
| --- | --- |
|  |  |
| The caller does not commit in answering whether the patient is conscious, saying “yes” at one point, “no” at another and “I can’t tell” at another. The dispatcher asks if she can speak with the patient. The caller says, “No, there’s no way he can talk to you.” | If the caller reports that the patient can’t speak, it indicates the patient is most likely not conscious. Mark the “No” circle, coding the patient as not conscious. |
| A caller says the patient is in a seizure. The seizure then stops, and the caller reports that the patient “is snoring like he’s in a deep sleep and he won’t wake up.” | A patient who “won’t wake up” should be classified as not conscious. Mark the circle next to “No”, coding the patient as not conscious. |
| The caller reports the patient wouldn’t wake up a minute ago, but now appears to be “getting better.” The dispatcher tells the caller to shake the patient’s shoulders to see if the patient responds. The caller says he moaned and pushed her arms away. | A patient who makes purposeful movement (pushing the caller’s arms away) is demonstrating conscious intent and should be coded as conscious. Mark the circle next to “Yes”. |

# Breathing addressed?

- Yes
- No
- Unknown
- N/A
- Not relevant

1: If signs of breathing are addressed by either dispatcher or caller or the caller says that the patient is in cardiac arrest.

2: If breathing is not addressed.

N/A: When in doubt

Not Relevant: Dispatchers initiate CPR instructions without addressing breathing. For example in cases where caller say that the patient is dead.

# Abnormal breathing adressed

- Yes
- No

# Patient is breathing normally?

- Yes - If the caller reports that the patient is breathing normally.
- No - If the caller reports that the patient is not breathing.
- No – If the caller reports that the patient is not breathing normally.
  - Descriptions of abnormal breathing includes, but is not limited to, “gasping”, “gasping for air”, “gurgling”, “snoring”, “humming”, “moaning”, “breathing every once in a while”, and “shallow breathing”.
- Unknown
- N/A

**Definition:**

A patient is considered to be breathing normally if the caller reports the patient is breathing normally. A patient is considered to be not breathing if the caller reports the patient is not breathing. A patient is considered to be not breathing normally if (A) the caller reports abnormal breathing and/or (B) the Quality Assurance (QA) rater hears abnormal breathing and/or identifies it through the caller’s description of the patient’s breathing. Abnormal breathing is defined as breathing with a rate and/or character different from the victim’s normal breathing at rest.

**Description:**

A patient’s breathing status is a key indicator of whether he or she is in cardiac arrest. It can be difficult to get a clear answer on whether the patient is breathing normally. Callers often give contrary answers to this question at different times in the call. Agonal breathing is very common in cardiac arrest. Callers often use specific words or phrases to describe this kind of breathing. These descriptions include, but are not limited to, “gasping,” “gasping for air,” “gurgling,” “gargling,” “snoring,” “snorting,” “humming,” “moaning,” “groaning,” “breathing every once in a while” and “shallow breathing.” Type-appropriate CPR instructions should be given when a patient is deemed not breathing normally and not conscious.

**Instructions for Coding:**

Mark the circle next to the appropriate answer (“Yes,” No,” or “Unknown”) under “Breathing Normally?” on the “Dispatch: Patient” section of the QA form. In cases where callers describe agonal breathing or where the quality assurance rater hears agonal breathing, patients should be coded as not breathing normally.

| **Examples:**  The caller says her husband is drunk and that he keeps “gurgling and gasping for air.” | The descriptors “gurgling and gasping for air” indicate agonal breathing. Even if the caller suspects it’s just because her husband is drunk, mark the “No, not breathing normally” circle, coding the patient as not breathing normally. |
| --- | --- |
| The caller says his wife “seems to be breathing okay,” but the quality assurance rater hears a soft snoring sound in the background. The dispatcher does not hear it or hears it but does not identify it as abnormal breathing. | Mark the “No, not breathing normally” circle, coding the patient as not breathing normally. |

# Did dispatcher recognise need for CPR?

- Yes - If dispatcher actively orders or passively instructs bystanders to start CPR.
- No - If dispatcher does not order bystanders to start CPR.
- Unknown
- N/A

**Definition:**

 A dispatcher or call-taker recognizes the need for CPR when he or she indicates that CPR should be performed in the course of the call.

**Description:**

 The dispatcher recognizes the need for CPR when he or she says any of the following in connection with a response to the victim’s condition: “CPR,” “chest compressions,” “compressions,” “continuous chest compressions,” “CCR,” “rescue breaths,” “rescue breathing,” “ventilations,” or “rescue ventilations.” In some cases, the dispatcher might not say any of these but indicates recognition by starting CPR instructions. In such cases, the time to dispatch recognition of the need for CPR and the time to start of CPR instructions are the same.

**Instructions for Coding:**

 If the dispatcher indicates that he or she recognizes the need for CPR, mark the circle next to “Yes” under “Did dispatch recognize the need for CPR?”

 If the dispatcher does not indicate that he or she recognizes the need for CPR, mark the circle next to "No.”

 If it is not known whether the dispatcher indicated recognition of the need for CPR, mark the circle next to “Unknown.”

# Delayed recognition due to deviation from cardiac arrest protocol

- Yes – If cardiac arrest protocol is deviated at any time of the call
- No – Compliance to cardiac arrest protocol
- N/A

**Instructions for coding:**

If agonal breathing is misinterpreted and dispatcher instructs caller to put the patient in recovery position at any time of the call, mark the circle next to «Yes»

If recogition is delayed due to circumstances at scene, mark the circle next to «No».

# Time of recognition (MM:SS)

- Short answer text

**Definition:**

 The time dispatch recognizes the need for CPR is the time elapsed from the start of the call to the moment when the dispatcher or call-taker indicates that he or she realizes CPR should be performed.

**Description:**

If CA has been recognised before the call, time is set to 00:00.

Need for CPR is not recognised if instructions of CPR are mentioned by the dispatcher, and he/she then instructs bystander to check the patients breathing first. In this case, time of recognition is when the first compression is made by a bystander.

 Dispatcher and call-taker recognition of the need for CPR is the first of three key time intervals in the provision of pre-arrival CPR instructions.

 Dispatchers and call-takers indicate their recognition when they say any of the following in connection with a response to the patient’s condition: “Cardiopulmonary Resuscitation,” “CPR,” “chest compressions,” “compressions,” “continuous chest compressions,” “Hands-Only CPR,” “CCR,” “rescue breaths,” “rescue breathing,” “ventilations,” or “rescue ventilations.” In some cases, the dispatcher might not say any of these but indicates recognition by starting CPR instructions. In such cases, the time to dispatch recognition of the need for CPR and the time to start of CPR instructions are the same.

 If the dispatcher or call-taker indicates his or her recognition, but subsequently instructs the caller or rescuer either to “lift the patient’s chin and tilt his or her head back” and/or “to look, listen and feel for breathing,” the time to dispatch recognition of the need for CPR should be defined as the moment the dispatcher or call-taker indicates his or her recognition AFTER instructing the caller or rescuer to perform this formal breathing assessment.

**Instructions for Coding:**

 Enter in minutes (“MM”) and seconds (“SS”) the elapsed time from the start of the call (or in the case of a Transfer Call, the time elapsed from the moment the dispatcher or call-taker first addresses the caller) to the moment of dispatch recognition of the need for CPR.

| **Examples:** The dispatcher says, “We need to start CPR right away.” | Enter the time elapsed to the moment when the dispatcher says “CPR.” |
| --- | --- |
| The dispatcher says, “We need to start CPR” at 1 minute and 27 seconds into the call. She then instructs the caller to lift the patient’s chin, tilt his head back and to look, listen and feel for breathing. The caller performs this procedure. It takes 25 seconds, and at 1:52 the dispatcher says, “OK, let’s start compressions.” | Enter 1:52 as the time to dispatch recognition of the need for CPR |

# BLS competence addressed?

- Yes - If bystander's BLS competence is addressed by dispatcher.
- No - If bystander's BLS competence is not addressed by dispatcher.
- N/A

1: If previous experience, skills or received training is mentioned.
2: If BLS competence is not addressed.
88: If OHCA is not recognized
99: When in doubt

# CPR instructions started?

- Yes - Instructions start when dispatcher tells the bystander to “kneel by the patient’s side” or similar phrases
- No - If instructions are not started. Dispatcher instructing to get the patient to a hard, flat surface is not considered CPR-instructions.
- N/A

**Definition:**

 CPR instructions are directions dispatchers and call-takers provide to guide callers through the process of performing CPR, whether compression-only or conventional CPR (CPR with rescue breathing). Instructions are considered “started” if they are simply started, even if they are not finished.

**Description:**

 Instructions to get a patient to a hard, flat surface should not be considered the start of CPR instructions. In many protocols, instructions start when a call-taker or dispatcher tells the rescuer to “kneel by the patient’s side”. The moment when CPR instructions are considered started, however, may vary from one dispatch center to another according to language used in local protocols.

**Instructions for Coding:**

 If CPR instructions are started, mark the circle next to “Yes” under “CPR instructions started?”

 If CPR instructions are not started, mark the circle next to "No.”

 If it is not known whether CPR instructions were started, mark the circle next to “Unknown.”

| **Examples:**  A caller is ready to start CPR. The dispatcher begins instructions, saying, “Kneel by the patient’s side,” but the caller stops him abruptly, saying the patient is “waking up and is conscious now.” The dispatcher does not continue the CPR instructions he started.  Caller start CPR without instructions from dispatcher and no instructions are provided by the dispatcher throughout the call  Caller start CPR without instructions from dispatcher, but dispatcher gives instructions after CPR is started | Code as “Yes.” Although CPR instructions were stopped just after they were started in this example, they were still started  Coded as «No»  Coded as «Yes» |
| --- | --- |

# Is the dispatcher assertive or passive when providing CPR instructions?

- Assertive
- Passive
- Unknown
- N/A

**Instructions for Coding:**

Coded as «Assertive» if dispatcher tells callers what to do, e.g. “We need to start CPR” or “I need you to start CPR". Coded as «Passive» if dispatcher ask caller if he/she wants to do CPR instead of telling caller "We need to start CPR"

# Quality assessment

- Yes, when dispatcher comments on speed and depth
- No
- Unknown
- N/A (No CPR)

**Instructions for Coding:**

Coded as «Yes» if dispatcher is checking quality of CPR. E.g. «please count out loud with me», «are you pushing deep enough?» «push a bit faster/slower»

# Encouraging/motivating techniques in use

- Yes, if encouraging or motivating techniques are in use
- No, if no encouraging or motivating techniques are in use
- Unknown
- N/A

**Instructions for Coding:**

Coded as Yes, if encouraging or motivating techniques are in use, e.g. «keep on going», «you’re doing a great job», «the ambulance is on its way», Encouraging or motivating techniques must be ongoing throughout the call, it is not enough to just say «keep on going» once.

# Type of CPR

- 30:2
- CC, continous compressions (ventilations are not provided)
- Unknown
- N/A

**Instructions for Coding:**

If dispatcher starts instructions for compressions only, but changes to include ventilations after a while, code as “30:2”

# Time of chest compression instructions started (MM:SS)

- Short answer text

**Definition:**

 This is the time elapsed from the start of the call (or in the case of a Transfer Call, the time elapsed from the moment the dispatcher or call-taker first addresses the caller) to the moment when the dispatcher or call-taker starts CPR instructions.

**Description:**

 The time at which a dispatcher or call-taker starts CPR instructions is the second key time interval in the provision of pre-arrival instructions. This method for assigning this time will vary from dispatch center to dispatch center, depending on the wording of protocols. Instructions to get a patient to a hard, flat surface should not be considered the start of CPR instructions. In many protocols, instructions begin when a call-taker or dispatcher tells the rescuer to “kneel by the patient’s side.”

**Instructions for Coding:**

 Enter in minutes (“MM”) and seconds (“SS”) the elapsed time from the start of the call (or, in the case of a Transfer Call, the time elapsed from the moment the dispatcher or call-taker first addresses the caller) to the moment the dispatcher or call-taker starts CPR instructions.

| **Examples:**  The caller reports that she is ready to start CPR. The dispatcher says, “kneel by his side and put the palm of one hand in the center of his chest ,” at 2 minutes and 12 seconds. | Enter 2:12 as the time at which the dispatcher began instructions for CPR |
| --- | --- |

# Chest compressions started?

- Yes - If there is a clear sound of compression, if bystander counts loudly or actively say that they are doing compressions or if they think/know someone else is doing compressions.
- No - If none of the above occurred.
- N/A

**Definition:**

 Chest compressions are considered “started” if a rescuer does *any* chest compressions, even if the rescuer stops just after starting.

**Description:**

 Determining whether chest compressions are started can be difficult in a minority of cases. Rescuers don’t always count out their compressions, and sometimes their voices or the compressions themselves are inaudible.

**Instructions for Coding:**

 If chest compressions were started, mark the circle next to “Yes” under “Chest Compressions Started?”

 If chest compressions were not started, mark the circle next to "No.”

 If it is not known whether chest compressions were started, mark the circle next to “Unknown.”

| **Examples:**  Caller states he will start CPR, puts down the phone and dispatcher is not able to get in touch with the caller again | Coded as «Yes», chest compressions started |
| --- | --- |

# Time of first chest compression (MM:SS)

- Short answer text

**Definition:**

 This is the time elapsed from the start of the call (or in the case of a Transfer Call, the time elapsed from the moment the dispatcher or call-taker first addresses the caller) to the moment when the caller or rescuer delivers the first chest compression.

**Description:**

 The time to first compression is the third of three key time intervals in the provision of pre-arrival CPR instructions. The time is noted when the first compression is audible or the caller/rescuer indicates he or she has started compressions (i.e. by counting with dispatcher).

**Instructions for Coding:**

 Enter in minutes (“MM”) and seconds (“SS”) the elapsed time from the start of the call (or, in the case of a Transfer Call, the time elapsed from the moment the dispatcher or call-taker first addresses the caller) to the moment the caller or rescuer delivers the first chest compression. There are often calls in which the time to first compression must be carefully inferred or entered as “Unknown.”

| **Examples:**  The dispatcher finishes instructions for starting compressions, and the caller clearly counts out the first compression at 3 minutes and 23 seconds into the call. | Enter the time elapsed to first compression as 3:23. |
| --- | --- |
| The dispatcher finishes instructions for CPR at 2 minutes and 50 seconds into the call and tells the caller to count the compressions out loud. The caller doesn’t count, however, and, eight seconds later, at 2:58, the dispatcher asks, “Are you doing the compressions?” The caller says, “Yes.” The dispatcher then reminds the caller to count out loud, and the caller begins: “1, 2, 3 …” | In this scenario, it becomes clear that the caller is doing CPR at 2:58 seconds (the caller says, “Yes” when asked if he’s doing compressions.) The dispatcher told him to count out loud at 2:50. Since 8 seconds later the caller said he had been doing compressions, it can be reasonably inferred that the first compression occurred somewhere between 2:51 and 2:55. In the absence of more perfect information, enter the elapsed time as 2:53, the midpoint between 2:51 and 2:55. |
| The dispatcher finishes instructions for CPR and tells the caller to count out loud at 1:46. The caller doesn’t count, but the first of a string of audible compressions occurs at 1:49. | Enter the time elapsed to first compression as 1:49. |

# Was an AED addressed

- Yes - If an AED or similar wording referring to an AED is mentioned by either caller or dispatcher
- No - If an AED or similar is not mentioned

N/A

1: If an AED or similar wording referring to an AED is mentioned by either caller or dispatcher.
2: If an AED is not mentioned
88: If OHCA is not recognized
99: When in doubt

# Was an AED connected to the patient?

- Yes - If the caller actively says that he/she or someone else has connected the AED to the patient, or it is clearly heard that the AED is on and gives instructions to the bystander.
- No - If none of the above occurred.
- N/A

# Did the AED deliver a shock to the patient?

- Yes - If the caller actively says that the AED has delivered shock/shocks, or it is clearly heard that the AED is giving instructions to deliver a shock.
- No - If none of the above occurred.
- N/A - If you answered no or N/A to number 20 or are in doubt.

# Barriers delaying or hindering CPR?

- Hang up phone - When the caller disconnects from the dispatcher processing the call.
- Language barrier - When the caller and dispatcher do not speak the same language and therefore can't communicate effectively.
- Caller left phone - When the caller leaves the phone for purposes other than rendering aid to the patient after speaking to the dispatcher.
- Caller not with patient - When the caller is speaking from a location that prohibits the caller’s physical assessment of the patient.
- Overly distraught - When a caller’s highly-distressed emotional state delays or prevents him or her from taking CPR instructions and/or performing CPR.
- Caller refused - When caller refuses for reasons other than a physical inability to perform CPR.
- Caller couldn't move patient - When a caller reports his or her inability to move the patient from an unsuitable location for CPR (e.g., toilet or bed).
- Patient's status changes - When a patient initially thought to be in cardiac arrest presents indication that he or she is not in cardiac arrest.
- Patient obviously dead - Caller conveys that patient is deceased. Caller provides sufficient evidence to the dispatcher in support of that conclusion (e.g. rigor mortis, mottled skin, decomposition, foul odor).
- DNR order.
- Other - Any barrier apart from those defined above that prevents the start of CPR instructions and/or bystander chest compressions.
- No barrier
- N/A (When CPR is not started)

**Definitions:**

 Barriers to CPR are defined as obstacles that prevent the start of dispatch-directed, bystander chest compressions. They include:

o Hang up phone: This is when the caller disconnects from the dispatcher or call-taker processing the call.

o Language barrier: This when the caller and dispatcher do not speak the same language and therefore cannot communicate effectively.

o Caller left phone: This is when the caller leaves the phone for purposes other than rendering aid to the patient after speaking with the dispatcher or call-taker.

o Caller not with patient: This is when the caller is speaking from a location that prohibits the caller’s physical assessment of patient.

o Overly distraught: This is when a caller’s highly-distressed emotional state delays or prevents him or her from taking CPR instructions and/or performing CPR.

o Caller refused: This is when a dispatcher or call-taker suggests or instructs CPR and a caller refuses for reasons other than a physical inability to perform CPR.

o Couldn’t move patient: This is when a caller reports his or her inability to move the patient from an unsuitable location for CPR (e.g., toilet or bed).

o Patient status change: This is when a patient initially thought to be in cardiac arrest presents indication that he or she is not in cardiac arrest.

o Obviously dead: Caller conveys that patient is deceased. In this case, the caller provides sufficient evidence to the dispatcher in support of that conclusion (e.g. rigor mortis, mottled skin, decomposition, foul odor).

o Other: Any barrier apart from those defined above that prevents the start of CPR instructions and/or bystander chest compressions

**Description:**

 Barriers to CPR are important to track because the recurrence of given barriers can point the way to protocol changes addressing high-frequency obstacles. For example, a common barrier is that rescuers can’t move a patient from a bed to a suitable location where compressions could be effective. Knowing this, managers and medical directors can experiment with protocol language and procedures to help rescuers solve this problem.

 Multiple barriers can delay or prevent the start of CPR in any one call.

**Instructions for Coding:**

 Check the box next to the appropriate item under “Barriers to CPR” according to the definitions above.

11

| **Examples:**  The caller, a native Spanish speaker, speaks and understands English poorly. The dispatcher knows little Spanish, but is able to get the caller to do CPR after several minutes of trying to clarify his instructions. | Code as a delay to start of CPR resulting from “Language barrier” |
| --- | --- |
| The dispatcher tries to calm a hysterical caller, but the caller screams and then leaves the phone. The caller is heard screaming in the background until EMTs arrive. | Code as “Overly distraught” and  “Caller left phone” |
| A dispatcher tells the caller that she needs to start CPR and that he will help her. The caller refuses, however, saying she has hurt her back and that there is no way she can get the patient from the bed to the floor. | Code as “Other” (and what that “other” barrier was: physical inability). The caller has refused to take CPR instructions but for reasons owing to a physical inability to perform (her bad back).  Code as “Couldn’t move patient” |
| The patient appears to be unconscious in the back yard, but the caller is on a landline phone on the second floor of the house. The caller is thus not able to physically assess the patient’s status. | Code as “Caller not with patient” |
| The caller reports that the patient is not conscious and not breathing normally. The dispatcher starts instructions for CPR, but the patient opens his eyes and begins to mumble and deliberately starts rubbing his head. The dispatcher recognizes the patient is conscious and discontinues CPR instructions. | Code of “Patient status change” |
| The caller indicates that the patient is not conscious and not breathing normally. The dispatcher starts instructions for CPR, but the caller subsequently says the patient is “blue, cold and stiff as a board.” The dispatcher discontinues CPR instructions. | Code as “Obviously dead” |

# Was CA witnessed by another person?

- Yes - If the caller actively says that he/she or someone else saw the patient collapse, or if the caller or someone else heard the patient collapse and were by their side within seconds.
- No - If none of the above occurred.
- N/A

# Call continued until EMS arrival

- Yes - If it is addressed or clearly heard, that the ambulance personnel is by the patient
- No - If the call is terminated before the ambulance personnel arrives at the side of the patient, or if the medical dispatcher sees that the ambulance personnel has pushed “arrived” and ends the call, or the sirens is heard in the recording and call is ended.
- N/A

# Caller gender

- Female
- Male
- Other
- N/A

# Caller relation to patient

- Known patient - If caller is family (Mother, father, grandparents, uncle, aunt, cousin etc.), in-laws, friends, colleagues etc.
- Unknown patient – Caller does not know the patient prior to OHCA incident.
- N/A

# Healthcare professional

- Yes - If **caller** has healthcare education/background regardless of level (MD, nurse, paramedic, emergency medical technician, Social - and Health Service Helper/assistant, social worker at institutions, any of the above in training)
- No
- N/A

# Was caller alone at time of call?

- Yes
- No
- N/A

Yes: If no one else is heard.
No: If anyone else is heard or communicated to, it is addressed that the caller is not alone.
N/A: When in doubt.
